# Role of Insulin in Health and Disease: An Update

**DOI:** 10.3390/ijms22126403

**Published:** 2021-06-15

**Authors:** Md Saidur Rahman, Khandkar Shaharina Hossain, Sharnali Das, Sushmita Kundu, Elikanah Olusayo Adegoke, Md. Ataur Rahman, Md. Abdul Hannan, Md Jamal Uddin, Myung-Geol Pang

**Affiliations:** 1 Department of Animal Science & Technology and BET Research Institute, Chung-Ang University, Anseong 17546, Korea; shohagvet@gmail.com (M.S.R.); adegokeelkanah@gmail.com (E.O.A.); 2 ABEx Bio-Research Center, East Azampur, Dhaka 1230, Bangladesh; kshrumky2010@gmail.com (K.S.H.); sharno.mou13@gmail.com (S.D.); sushmitakundu150729@gmail.com (S.K.); ataur1981rahman@hotmail.com (M.A.R.); hannanbmb@bau.edu.bd (M.A.H.); hasan800920@gmail.com (M.J.U.); 3 Department of Pathology, College of Korean Medicine, Kyung Hee University, Seoul 02447, Korea; 4 Department of Biochemistry and Molecular Biology, Bangladesh Agricultural University, Mymensingh 2202, Bangladesh; 5 Graduate School of Pharmaceutical Sciences, College of Pharmacy, Ewha Woman’s University, Seoul 03760, Korea

**Keywords:** insulin, health, disease, homeostasis, regulation, glucose

## Abstract

Insulin is a polypeptide hormone mainly secreted by β cells in the islets of Langerhans of the pancreas. The hormone potentially coordinates with glucagon to modulate blood glucose levels; insulin acts via an anabolic pathway, while glucagon performs catabolic functions. Insulin regulates glucose levels in the bloodstream and induces glucose storage in the liver, muscles, and adipose tissue, resulting in overall weight gain. The modulation of a wide range of physiological processes by insulin makes its synthesis and levels critical in the onset and progression of several chronic diseases. Although clinical and basic research has made significant progress in understanding the role of insulin in several pathophysiological processes, many aspects of these functions have yet to be elucidated. This review provides an update on insulin secretion and regulation, and its physiological roles and functions in different organs and cells, and implications to overall health. We cast light on recent advances in insulin-signaling targeted therapies, the protective effects of insulin signaling activators against disease, and recommendations and directions for future research.

## 1. Introduction

Insulin, a hormone composed of 51 amino acids, plays important roles in glucose homeostasis, cell growth, and metabolism. The isolation and purification of insulin in Toronto from 1921–1922 was pioneered by Dr. Frederick Banting [1]. Since its discovery, researchers have intensified efforts towards improving the quality of insulin. The discovery of insulin has also triggered the discovery of other hormones such as glucagon [2]. Insulin, upon discovery, represents lifesaving therapeutic for people suffering from diabetes. This hormone was previously believed to be solely produced by β cells of the pancreas; however, recent evidence has shown that low concentrations are also found in certain neurons of the central nervous system [3]. Although the biosynthesis and secretion of insulin are controlled by circulating glucose levels, the concentrations required to initiate the two processes differ [4,5]. While glucose concentrations above 5 mM are required to initiate insulin secretion, fluctuations within 2 mM to 4 mM stimulate its biosynthesis [4]. The metabolism of glucose is triggered by food intake, leading to simultaneously increased β cell insulin production and decreased α cell glucagon secretion to bring serum glucose levels back to normal [5]. Following secretion, insulin systemically circulates and is distributed to hepatocytes, which are prompted to store glucose in the form of glycogen. Skeletal muscle cells and adipocytes, the other major targets of circulating insulin, also take up glucose, thereby reducing the blood glucose concentration to baseline [6]. As with other protein hormones, insulin triggers glucose uptake, skeletal muscle protein synthesis, glycogenesis, and lipogenesis via the tyrosine kinase receptor pathway [7,8]. The insulin receptors present in the plasma membrane act enzymatically to transmit phosphates from ATP to tyrosine residues on intracellular target proteins [8]. Following the binding of insulin to the α subunits, the β subunits phosphorylate and, consequently, activate the receptor’s catalytic function [8]. The activated receptor also phosphorylates several intracellular proteins that regulate the metabolic activities of insulin, cell growth, and cell differentiation-related gene expression [9,10].

To date, the main focus of research has been to investigate the role of insulin in the onset and progression of pathological conditions and chronic diseases, such as diabetes. The literature shows that insulin deficiency makes it impossible for cells to use glucose as an energy source [6]; consequently, high-glucose concentrations in the bloodstream lead to a condition known as hyperglycemia [6]. Prolonged hyperglycemia leads to diabetes mellitus and can cause health complications such as nervous system damage and dysfunction of the eyes and kidneys. Similarly, a cell’s inability to use glucose as an energy source that results from the lack of insulin can trigger reliance on fat stores as the only energy supply. The continuous dependence on fat may induce the release of ketones into the bloodstream and lead to the chronic condition of ketoacidosis [11]. 

In addition to its role in diabetes, the recent literature indicates that insulin acts on several key organs in the body, including the brain, heart, kidney, bone, skin, and hair follicles, to perform important physiological roles. Insulin aids bone formation and attenuates osteoporosis-related inflammation [12], acts on the central nervous system [13,14], and performs pro- and anti-atherogenic functions in the vascular system [15]. Recent advances in insulin research have led to insulin-signaling targeted therapies and insulin-signaling activators being used as protective measures against several diseases. Clinical and laboratory studies have indicated that metformin, an insulin-receptor activator, has properties that protect the kidneys from injury [3]. Similarly, sulfonylurea, through its activities on enhanced insulin secretion via their actions on pancreatic β cells, augmented insulin secretion [4]. Currently available forms of insulin include insulin mixtures, concentrated insulins, and insulins with alternate routes of administration, providing several options for people living with diabetes. Exogenous insulins are now available in the form of rapid-acting, short-acting, intermediate-acting, and long-acting [16]. This article provides an updated review of insulin secretion and regulation, its physiological roles in body organs, the health consequences of insulin deficiency, and recent advances in insulin-signaling targeted therapies.

## 2. Regulation of Insulin Secretion

The physiology of insulin-producing cells is fundamental to understanding the regulation of insulin secretion. Insulin is a peptide hormone secreted by β cells of the pancreas. The human pancreas contains one to two million pancreatic islets [17] housing different endocrine cells, primarily insulin-secreting β cells, glucagon-producing α cells, and somatostatin-secreting δ cells [18]. Although islets compose only 1–2% of the human pancreas, they receive up to 10% of the total pancreatic blood supplies [19,20]. Generally, insulin is released after ingesting glucose in a process named glucose-induced insulin stimulation. This process requires both the intracellular uptake and metabolic degradation of ingested glucose [19,21]. In human β cells, glucose transporter 1 (GLUT1, encoded by SLC2A1) and GLUT3 (encoded by SLC2A3) are the prominent glucose transporters, whereas GLUT2 (encoded by SLC2A2) has been reported as a major glucose transporter in rodent [22,23]. This difference could be attributed to the differences in K_m_ values of different isoforms of glucose transporters [19]. 

The phosphorylation of glucose by the enzyme glucokinase (GCK) is the first step in glucose metabolism. Glucose phosphorylation by GCK is related to insulin secretion; therefore, GCK gene dysfunction or aberration leads to decreased glucose-mediated insulin release and glucose intolerance or diabetes [24]. A major understanding of insulin secretion is derived from the research using rodent models, whereas few studies have been described in humans [19]. 

In nondiabetic donors, an increase in glucose concentration from 1 mM to 6 mM raises the rate of glucose oxidation threefold (as determined by measuring the levels of C_14_O_2_ produced from uniformly C^14^-labeled glucose), and an approximately 25% acceleration takes place when the concentration of glucose is increased to >12 mM [25]. About one-tenth of the ingested glucose enters into glycolysis, which occurs through mitochondrial oxidation in human islets [25], but the ultimate destination of the rest of the ingested glucose needs to be further elucidated. 

Glucagon-like peptide-1 and glucose-dependent insulinotropic polypeptide, which are the incretin hormones of the gastrointestinal tract, largely promote nutrient-induced insulin secretion, and they are highly crucial in the overall physiology of insulin secretion [19,26]. Research has indicated that incretins are capable of binding to G-protein-coupled receptors on β cell membranes and increase cellular 3′,5′-cyclic adenosine monophosphate (cAMP) levels and glucose-stimulated insulin secretion (GSIS) in the presence of higher glucose levels. Indeed, the action of incretins is somewhat resistant to diazoxide; therefore, it is independent of KATP channel closure [26]. As a consequence, cAMP induces an upsurge in the size/amount of readily releasable pools in a glucose concentration-dependent manner within insulin granule dynamics. It is worth noting that incretin peaks the activity of the β cells in the presence of active glucose concentrations in a Ca^2+^-independent manner, even in Ca^2+^-devoid conditions [27].

## 3. Insulin Signaling Pathways

**Figure 1:**
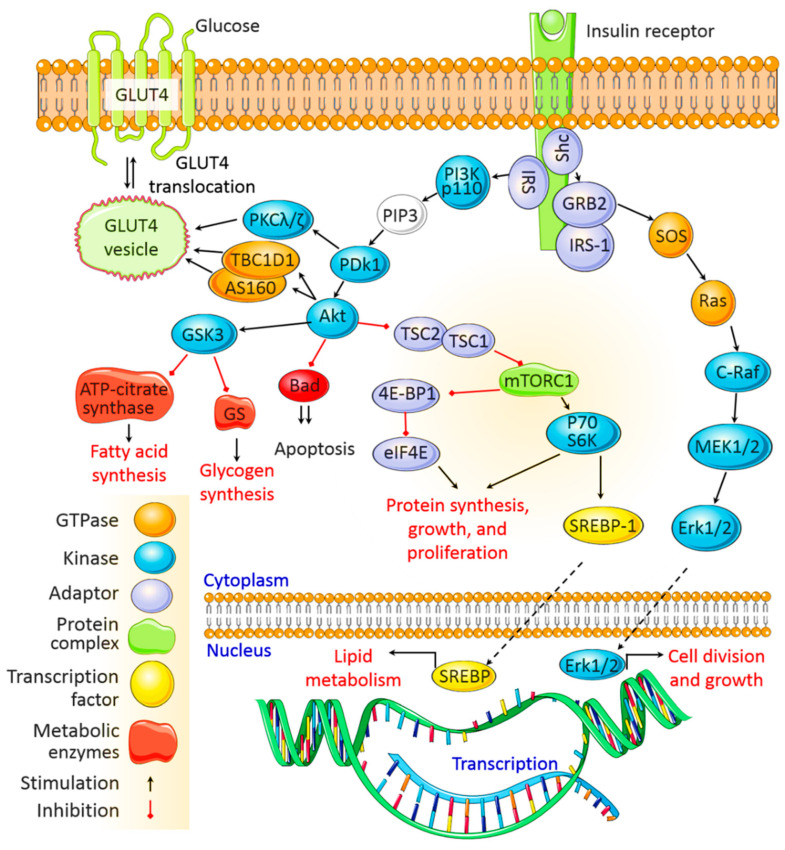
Classic insulin signaling pathway. Insulin regulates cellular functions and metabolic activity by binding to the insulin receptor. Core cellular processes downstream of the insulin signaling system include the PI3K/Akt and MAPK signaling pathways; details on these pathways are given in Section 3. IRS, insulin receptor substrate; IR, insulin receptor; PI3K, phosphoinositide3-kinase; MAPK, mitogen-activated protein kinase; PIP3, phosphatidylinositol 3,4,5-triphosphate; PDK1, 3-phosphoinositide dependent protein kinase-1; Grb2, growth factor receptor-bound protein 2; GSK, glycogen synthase kinase; GS, glycogen synthase; mTORC1, mammalian target of rapamycin complex 1; SREBP, sterol regulatory element-binding protein; glucose transporter 4, GLUT4; MEK, MAPK/Erk kinase; ERK, extracellular signal-regulated kinase.

After being secreted from pancreatic β cells and circulating through the body, insulin binds to insulin receptors (IRs) on target cell membranes. This results in the phosphorylation of insulin receptor substrate (IRS) and the subsequent activation of two primary signaling pathways, viz. the phosphoinositide3-kinase (PI3K)/protein kinase B (Akt) pathway and the mitogen-activated protein kinase (MAPK) pathway (Figure 1). 

### 3.1. The PI3K/Akt Signaling Pathway

The regulatory roles of insulin in cellular function and energy metabolism are largely mediated by the PI3K/Akt pathway [28]. Once activated by IRS, PI3K phosphorylates phosphatidylinositol 4,5-bisphosphate to produce phosphatidylinositol 3,4,5-triphosphate, which phosphorylates, and thus activates, 3-phosphoinositide dependent protein kinase-1 (PDK1). PDK1 then activates Akt, which mediates multiple cellular functions. Activated Akt phosphorylates glycogen synthase kinase to deactivate it and inhibits glycogen synthase and ATP-citrate lyase activity, thereby inhibiting glycogen and fatty acid synthesis, respectively. Akt also inactivates the mammalian target of rapamycin complex 1 to promote protein synthesis. In addition, Akt mediates cell survival by inhibiting the proapoptotic pathway, and it activates sterol regulatory binding proteins (SREBPs), which translocate to the nucleus to transcribe genes associated with fatty acid and cholesterol synthesis. The PI3K/Akt signaling pathway also regulates the translocation of the insulin-sensitive glucose transporter GLUT4 to the membrane of muscle and fat cells for glucose uptake. GLUT4 translocation involves the IR-facilitated phosphorylation of Cbl-associated protein (CAP) and production of the CAP:CBL:CRKII complex [28,29] (Figure 1).

### 3.2. The MAPK Signaling Pathway

The MAPK pathway is activated when IRS-1 binds to growth factor receptor-bound protein 2 (Grb2). SOS binds to Grb2 and then to Ras, causing GDP–GTP exchange and the activation of Ras. 

Activated Ras recruits c-Raf, which phosphorylates and activates MAPK/Erk kinase (MEK). MEK then phosphorylates extracellular signal-regulated kinase (Erk). Once activated, Erk is translocated to the nucleus, where its subsequent phosphorylation and transcriptional activation by transcription factors, such as ELK1, ultimately promote cell division, protein synthesis, and cell growth [28,29] (Figure 1).

## 4. Physiological Roles of Insulin

The major purpose of insulin is to regulate the body’s energy supply by balancing micronutrient levels during the fed state [30]. Insulin is critical for transporting intracellular glucose to insulin-dependent cells/tissues, such as liver, muscle, and adipose tissue. Any imbalance in exogenous energy supplies results in the breakdown of fats stored in adipose tissue and eventually accelerates insulin secretion. In the following sections, we discuss the major role of insulin in regulating several insulin-dependent tissue/organ functions. 

### 4.1. Role of Insulin in the Regulation of Liver Function

**Figure 2:**
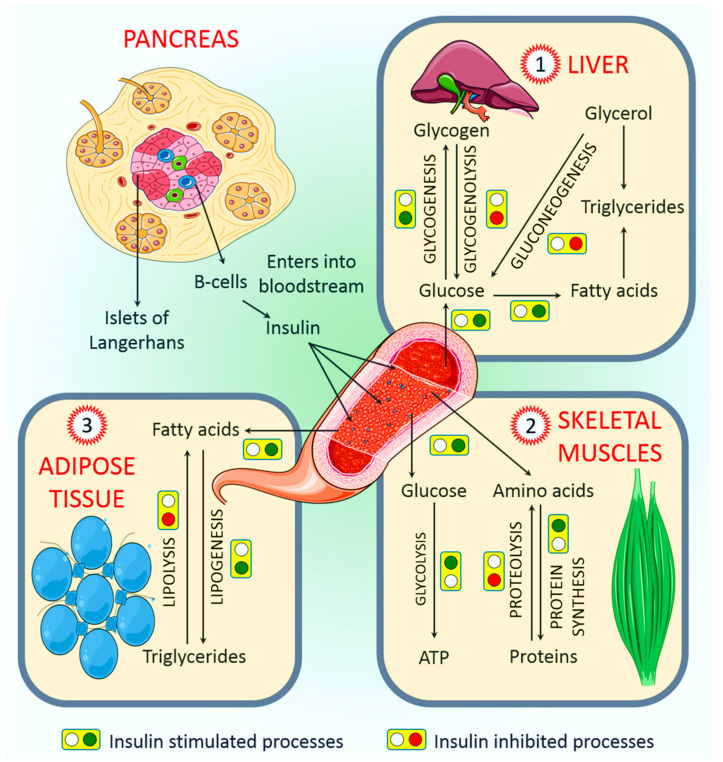
Major physiological roles of insulin in the liver, adipose tissue, and skeletal muscles. After production and release from pancreatic β cells, insulin enters the bloodstream to ultimately reach all other organs. In the liver, insulin helps promote the transport of glucose from the blood into hepatocytes, where it is further converted to glycogen, fatty acids, and triglycerides. In the skeletal muscles, insulin facilitates the uptake of glucose and amino acids from the bloodstream. The amino acids are subsequently used for functional protein synthesis, while glucose is mostly utilized in glycolysis to produce energy in the form of ATP. Glucose may also be converted to the glycogen that is mostly stored as energy for times of deficit. Insulin stimulates adipose tissue uptake of fatty acids, which are later converted into triglycerides and used as long-term energy stores. It is important to note that each of the steps/processes regulated by insulin in the figure are reversible. Whenever insulin stimulates the processes, they are generally irreversible.

The liver is the primary organ of insulin action. Among the many important functions of insulin, nutrient homeostasis, i.e., synthesizing glycogen from glucose and the conversion of excess glucose into fatty acids and precursor triglyceride (TAG), are the most crucial [31]. A search of the literature indicates that insulin upregulates glucose-utilizing activity via accelerated hepatic glucose utilization, glycolysis, and glycogenesis, and it downregulates glucose production by suppressing net glucose production, gluconeogenesis, and glycogenolysis [32]. Simultaneously, elevated blood glucose levels return to baseline via glycogenesis due to accelerated glucose uptake by adipose tissue and skeletal muscle [33] (Figure 2). In a healthy individual, a maximum one-fifth overturn of hyperinsulinemia has been reported to occur through insulin-driven gluconeogenesis in the liver, which also suppresses glycogenolysis almost completely [34]. Insulin can produce glucose for catabolic reactions through a process named gluconeogenesis, which acts directly on the liver but indirectly on other tissues [35,36]. Through the PI3K/phosphorylation of the Akt/IRS-1 pathway (Figure 1), insulin can inhibit hepatic gluconeogenesis while improving glycogen synthesis [36,37]. Although the precise mechanism by which insulin regulates hepatic function is yet to be investigated, it has been suggested that insulin acts directly and indirectly on the liver. Directly, it can bind with hepatic insulin receptors and subsequently activates insulin signaling pathways in the liver, which has been demonstrated both in vitro and in vivo experimental models [38,39,40,41]. On the other hand, indirect insulin action is mostly regulated by the reduction in pancreatic glucagon secretion [42], the inhibition of fat lipolysis [43], and the influence of overall hypothalamic insulin signaling [44], which subsequently affects hepatic glucose production. Although evidence indicated both direct and indirect effects of insulin on the liver, it has been suggested that the control of the liver function is largely indirect [45].

### 4.2. Role of Insulin in the Regulation of Skeletal Muscle Function

Insulin performs several important functions in all body parts, and skeletal muscle is no exception. Skeletal muscle is one of the most dynamic tissues of the human body, and it represents almost half of the body’s weight and two-thirds of its protein [46]. This muscle comprises bundles of highly structured muscle fiber/myofibers, with every myofiber, which contains several myofibrils, representing an individual muscle cell. Approximately 70% of the glucose is used by skeletal muscle from the whole-body glucose uptake [47], and the rest is used by the liver through an insulin-dependent mechanism. Postprandial hyperglycemia encourages the pancreas to secrete insulin, and an increase in plasma insulin concentration triggers the uptake and use of glucose by skeletal muscle [48,49,50]. Both epidemiological and experimental observations indicate that individuals with type 2 diabetes are associated with poor muscle strength and function [51]. Given that skeletal muscle is a major site for glucose disposal, quantitative declines in muscle volume in patients with type 2 diabetes might adversely affect overall glucose metabolism; thus, insulin therapy could improve optimal glucose targets [52]. 

Like other parts of the body, the muscle needs a continuous source of energy to work efficiently, and carbohydrates and fats are the main energy sources used by muscle cells to produce ATP [53]. Amino acids may also be used as an energy supply by muscle, but this depends on several factors [46]. After glucose is consumed, the plasma glucose concentration modulates insulin secretion by pancreatic β cells, which creates hyperinsulinemia. This leads to a decline in the plasma free fatty acid (FFA) concentration while decreasing lipid utilization. At the same time, insulin stimulates skeletal muscle to take up glucose by activating several enzymes [50] (Figure 2). In skeletal muscle control, glucose uptake and energy metabolism increase GLUT4 [54,55]. Insulin also controls the amount of branched-chain amino acids, non-esterified fatty acids, plasma glucose, and muscle mitochondrial ATP production [56].

### 4.3. Role of Insulin in the Regulation of Adipose Tissue Function

Every living organism needs energy to survive and has evolved to store extra energy when there is an abundance of food. This is an important physiological adaptation that increases the chances of survival during starvation. In some organisms, specialized cells store extra energy in the form of lipids, and in multicellular organisms, adipose tissue is responsible for the lipid storage [57]. Adipose tissue is found in every part of the body, which makes this tissue distinguishable from other tissues [58]. As mentioned earlier, approximately 70% of the glucose consumed is used by muscles as an energy source. Adipose tissue is responsible for almost one-tenth of the insulin for whole-body glucose uptake [59]. Therefore, the biological features of adipose tissue from different sites could play a significant role in the onset and progression of metabolic derangements, particularly in obese individuals. It has been reported that insulin regulates several aspects of adipose cells’ functional development and differentiation [60]. Adipose tissue is primarily independent of glucose uptake; however, it is dependent on the amount of FFA, which is liberated into the bloodstream by insulin for use by organs such as the heart [59]. It has been demonstrated that the TAG concentration is the main factor affecting the production of insulin [61] (Figure 2). However, further studies are needed to understand the molecular basis of adipose tissue response to insulin as it is highly relevant to the pharmacotherapy of diabetes and weight gain.

### 4.4. Other Major Physiological Roles

#### 4.4.1. Endothelium and Vasculature

The endothelium, which forms the inner layer of blood and lymph vessels, is considered a highly active part of the body involved in several pathophysiological processes, such as leukocyte adhesion, the control of vasomotor tone, inflammation, and barrier function [62]. Insulin is thought to be important for several functions relating to the endothelium, among which endothelium dysfunction is the most discussed [63]. Generally, endothelial dysfunction indicates events such as reduced nitric oxide (NO) bioavailability, increased oxidative stress mediated by elevated reactive oxygen species (ROS) production, the expression of pro-inflammatory and pro-thrombotic factors, and abnormal vasoreactivity [63]. This condition may predispose to increase susceptibility to atherosclerosis, coronary heart disease, and hypertension [64]. The binding of the insulin into the endothelial insulin receptors triggers two major signaling cascades mediated by PI3K and MAPK subsequently involved in these dysfunctional processes by regulating NO production [65]. Indeed, an in-depth understanding of insulin’s role in both the physiology and physiology of endothelial cells is essential for developing novel approaches for treating and preventing cardiovascular complications [66]. 

#### 4.4.2. Brain

Initially, the brain was considered insulin insensitive because glucose uptake in this organ is unaffected by insulin [67,68]. However, growing evidence suggested that insulin can increase glucose uptake in the spinal cord tissues and some brain regions, such as the choroid plexus, the pineal gland, and the pituitary [69,70,71]. In addition to glucose metabolism and the energy balance in the brain, insulin is reported to control other vital physiological functions, such as neuronal plasticity, memory processing, and cognition [72,73,74]. Insulin has been shown to decrease hepatic glucose production (HGP) in the central nervous system when delivered in an intracerebroventricular manner [44]. While acting on HGP, insulin can influence certain gluconeogenic enzymes by affecting the central nervous system [70,71]. In another study, it has been reported that intranasal administration of single-dose (160 IU) insulin declines food intake in healthy males but not in women [75]. Simultaneously, a significantly improved memory process is noticed only in women rather than men [75]. Although the precise mechanism of these effects is unknown, it has been suggested that the fundamental gender difference of the central nervous system might respond differentially to the acute insulin administration, and subsequently regulate energy homeostasis and memory functions in a sex-dependent manner [75]. Convergent with these observations, earlier studies suggested that insulin transport into the brain is equally affected both obese men and women. However, the risk of developing dementia is particularly higher in women rather than men [76,77]. Further, the ameliorative effects on the cognitive functions have been reported in healthy subjects following acute and chronic (8 weeks, 160 IU per day) intranasal administration of insulin [78,79]. Therefore, depending on the evidence described above, insulin plays a very important role in brain function and might be an excellent therapeutic compound for treating both obesity and Alzheimer’s disease, as suggested by other contemporaries [80,81,82].

#### 4.4.3. Kidney

The initial evidence of insulin actions in the kidney has been suggested in the 1950s [83]. Insulin involves various metabolic processes, including kidney homeostasis [84]. Recently, researchers have begun exploring the physiological importance of kidney IR. In diabetic and insulin-resistant rat models, the decreased expression of IRs and phosphorylated IRs was seen in renal epithelial cells [85,86]. A high-fat diet reduced IR expression in the kidney cortex of mice [87], and type 1 diabetic rat models and type 2 diabetic patients showed decreased expression of IRs in the kidneys [85]. Moreover, various kidney epithelial cell-specific deletions of IRs resulted in altered renal and systemic metabolism in mice [84]. By interacting with angiotensin type 1 receptors in the renin–angiotensin system, angiotensin II (Ang-II) mediates various biological effects in many tissues, including those of the kidney [88]. Ang-II hinders the insulin-mediated activation of PI3K signaling, leading to insulin resistance [89]. Furthermore, insulin is a critical regulator of glucose metabolism in the kidneys. Hyperglycemia is responsible for the development of diabetic kidney diseases (DKDs). Therefore, the progression of kidney diseases, including DKD, could be slowed or reversed by controlling hyperglycemia with insulin therapy [90].

#### 4.4.4. Bone

Another important physiological role of insulin is that it promotes bone development [91]. Bone formation may be regulated by insulin signaling through osteoblast development and bone resorption by osteoclasts [91]. According to experiments with mice, these cells express IRs on their surface [92]. That said, IRs in osteoblasts are needed for the proliferation, survival, and differentiation of osteoblasts. In another in vitro study, it has been reported that insulin is an anabolic agent capable of increasing the rate of osteoblast proliferation, collagen synthesis, and alkaline phosphatase production, and it facilitates glucose uptake and subsequently inhibits the activities of osteoclasts [93]. Additionally, insulin-deficient models displayed a decline in mineralized and osteoid surface areas due to altered mineral apposition rates and osteoblast activity [93], and epidemiological investigations have further reinforced this finding. Patients with type 1 diabetes were found to have low bone mineral density and a higher risk of fractures than their non-diabetic counterparts [15,93]. 

#### 4.4.5. Skin and Hair Follicles

Hair follicles are important parts of the body and, as a highly active organ, they require a sophisticated regulatory micro-environment with an ample supply of oxygen and nutrients [94]. Therefore, chronic decreases in oxygen and nutrient supplies due to hyperglycemic conditions may lead to follicular damage, which results in the alteration of regular hair growth (e.g., thinning, fragility, sparseness of hair, and decline in hair growth) [13,14,95]. The corresponding relationship between insulin action and the hair follicle is not fully understood. However, certain skin conditions, such as acrochordon and acne, have been linked with insulin action [96,97]. Acne is considered a symptom of many diseases linked with IRs, whereas individuals suffering from acrochordon have shown greater carbohydrate metabolic impairment than healthy individuals [98]. Those who consume low-insulinotropic paleolithic diets exhibit much lower insulin/IGF-1 signaling, which is linked to a reduced occurrence of acne [99,100,101]. Another skin disease, psoriasis, is connected to other metabolic diseases, including obesity and metabolic syndrome, and there is a link between psoriasis and IR through the common factor of adipose tissue [12,102]. Insulin sensitivity is reportedly deregulated in individuals who have psoriasis based on their leptin and adiponectin levels, which regulate insulin sensitivity/activity through the inflection of insulin signaling and the molecules associated with glucose and lipid metabolism [103,104,105].

## 5. Role of Insulin in Pathology

### 5.1. Insulin Deficiency

Nutrient availability plays an important role in the secretion and functional regulation of insulin. The excessive consumption of fatty foods can alter mitochondrial physiology by enhancing the excessive ROS production that impairs insulin action. It has been found that insulin-resistant individuals in an aerobic state during exercise can stimulate both mitochondrial biogenesis and efficiency concurrently with insulin activity [106]. People over 30 years of age with type 1 diabetes, as defined by severe insulin shortage, have similar clinical and biological features to younger people, but the condition is frequently not recognized [107]. The overproduction of glucose and the buildup of lipids should be anticipated in the livers of patients with obesity and insulin resistance [108]. Therefore, both intrahepatic and extrahepatic pathways mediate insulin’s control of glucose and lipid metabolism, and the interactions between these pathways control insulin signaling [109]. Direct hepatocyte insulin signaling is essential for lipogenesis but unessential for suppressing glucose production [110]. Pathologically, both insulin resistance and insulin deficiency alone can change plasma glucose levels [111]. Lengthening the action time of basal insulin and restricting peaks of fast-acting insulin can be beneficial for individuals with diabetes. Different transport systems may make the regular use of insulin more acceptable to patients and may have other advantages, such as aiding in attaining better glycemic control [112]. Closed-loop systems, or artificial pancreases, have shown safety and glycemic benefits [113]. Short-term insulin glargine administrations are partially beneficial for those with a β cell phenotype, whereas the long-term replacement of insulin by isogenic islet transplantation promotes the formation of more mature β cells [114]. Increased insulin resistance is an additional factor that can work in unison with other factors and may be important in the pathogenesis of diabetic microvascular complications [115]. Research has shown that if blood glucose remains high despite substantial insulin levels, the action of the hormone must be defective [116]. The absence of first-phase insulin reactions to intravenous glucose has long been considered an initial sign of β cell dysfunction and has some anticipative importance for the subsequent development and progression of diabetes [117].

### 5.2. Hyperinsulinemia

In hyperinsulinemia, the amount of insulin in the blood is higher than usual. The hyperinsulinemic state is characterized by damaged myocardial insulin signaling, mitochondrial dysfunction, endoplasmic reticulum stress, altered calcium homeostasis, irregular coronary microcirculation, sympathetic nervous system dysfunction, initiation of the renin–angiotensin–aldosterone system, and immune response abnormalities. These pathophysiological alterations result in increased oxidative stress, fibrosis, hypertrophy, diastolic cardiac dysfunction, and eventual systolic heart failure, and it is suggested that hyperinsulinemia may be the common element accounting for the association between obesity and type 2 diabetes [108]. The reference range for hyperinsulinemia is normally decided based on fasting glucose levels, including 5–13 μU/mL, ≤30 μU/mL, and 18–173 pmol/L (3–28 μU/mL) REF. Obesity and type 2 diabetes are classic states of insulin resistance [118]. Insulin resistance regulates insulin secretion, which ultimately leads to hyperinsulinemia [119], and hyperinsulinemia is associated with increased morbidity and mortality from cardiovascular complications in patients with obesity [107,120]. Generally, the main cause of hyperinsulinemia is insulin resistance, which the pancreas compensates for by producing more insulin. However, it can sometimes be caused by a rare tumor of pancreatic insulin-producing cells (insulinoma) or excessive numbers or growth of these cells (nesidioblastosis). This condition also leads to low blood sugar [105]. 

The direct effect of hyperinsulinemia includes type 2 diabetes, obesity, chronic inflammation, hypertriglyceridemia, and Alzheimer’s disease [121]. A study showed that increased dietary fatty acids stimulate intestine enterocyte incretin secretion, further elevating GSIS, even at low glucose levels; thus, fatty acids play a vital role in forming diabetic hyperinsulinemia [122]. The diabetic cardiomyopathy detected in hyperinsulinemic states is categorized by damaged myocardial insulin signaling, abnormal mitochondrial function, endoplasmic reticulum stress, impaired calcium homeostasis, abnormal coronary microcirculation, the activation of the sympathetic nervous system, the activation of the renin–angiotensin–aldosterone system, and maladaptive immune responses, and these pathophysiological alterations lead to oxidative stress, fibrosis, hypertrophy, diastolic cardiac dysfunction and, eventually, systolic heart failure [123]. Hyperinsulinemia in women suffering from polycystic ovarian syndrome is prognostic of health problems later in life, such as diabetes, cardiovascular disease, and infertility [124]. Chronic hyperinsulinemia has been shown to upregulate triglyceride (TG)-rich lipoproteins and to be a risk factor for atherosclerosis [31]. A healthy, balanced diet can help a person maintain a healthy weight and improve their overall bodily function. Specific diets can also prevent blood sugar spikes and facilitate the regulation of insulin levels. Diets that focus on glycemic control are beneficial when treating hyperinsulinemia: a diet low in simple carbohydrates can help patients to regulate their glucose levels. More importantly, glycemic control should be established very early in pregnancy to stop the initiation of fetal hyperinsulinemia [125].

### 5.3. Hyperglycemia

Hyperglycemia occurs when the blood glucose is greater than 125 mg/dL during fasting or 180 mg/dL 2 h postprandial [126,127,128]. Hyperglycemia has increased in recent years without an apparent difference between men and women, particularly due to decreased physical activities and increased obesity [129,130]. Islet dysfunction, reduced insulin secretion, decreased glucose utilization, and insulin resistance found in type 2 diabetes are factors contributing to the onset and progression of hyperglycemia [128,131,132]. Basal hyperglycemia occurs when there is a lower insulin-to-glucagon ratio owing to the increased production of glucose by the liver, whereas postprandial hyperglycemia arises due to a decrease in plasma insulin concentration or action that reduces glucose utilization in peripheral tissues [133]. The postprandial hyperglycemia status is defined by factors, such as the timing, quantity, and composition of the meal, carbohydrate content of the meal, and the resulting insulin production and inhibition of glucagon secretion [128,131,132]. When the fasting plasma glucose level is consistently ≥7 mmol/L (126 mg/dL) or when the 2 hours’ plasma glucose level following drinking a 75 g glucose load is consistently ≥11.1 mmol/L (200 mg/dL), diabetes is diagnosed or confirmed [128].

Meanwhile, clinical findings indicated that fasting or 2 h postprandial glucose levels below the diabetes cutoffs indicates cardiovascular disease [134,135]. Thus, a positive correlation exists between glucose level and cardiovascular disease risk [135]. Prolonged hyperglycemia could also lead to the onset of other life-threatening complications such as ketoacidosis and hyperglycemic hyperosmolar syndrome. Although their pathogenesis differs, the basic underlying mechanism for both disorders is a decrease in the effective net concentration of circulating insulin coupled with a concomitant elevation of counterregulatory hormones (e.g., glucagon, catecholamines, cortisol, and growth hormone) [136]. As unhealthy diets and a lack of physical activity also contribute to a global rise in the prevalence of obesity [137] and both type 1 and II diabetes [138], a lifestyle change could be a good companion to insulin-signaling targeted therapy in reducing hyperglycemia and associated diabetes.

### 5.4. Hyperlipidemia

The leptin receptor or obesity receptor (Ob-R), which belongs to the cytokine class I receptor family [139], mainly resides in β cells, and when activated, it suppresses insulin secretion, insulin gene expression, and influences the proliferation, apoptosis, and growth of β cells [140,141]. The function and survival of β cells are affected by tumor necrosis factor-alpha and interleukin-6 (IL-6) [142], and an increased amount of pro-inflammatory factors were found in the pancreatic islets during stress conditions with glucose and FFA [143,144]. An increase in plasma FFA is essential under fasting conditions to maintain basal insulin levels and normal insulin responses to glucose [145]; however, it can contribute to a situation named lipotoxicity, in which increased plasma FFA plays roles in sustaining insulin resistance and impaired β cell function [146,147].

Lipotoxicity was observed in an in vitro study that exposed pancreatic islets to an increased amount of FFA for a short period; the result was impaired glucose stimulated by insulin release [148]. It has been demonstrated that an increase in FFA can contribute to compromised β cell function via the intracellular accumulation of TG resulting from the activation of SREBPs or high levels of uncoupling protein 2, which is known to regulate cellular ATP levels [149,150]. FFA also stimulates the activation of NO synthase to induce insulin secretion [147,151]. NO has been reported to contribute to apoptosis by activating c-Jun N-terminal kinase, MAPK, and Akt inhibition [151]. Therefore, lipotoxicity can speed up the loss of β cell function and β cell mass, all of which frequently occur in individuals suffering from type 2 diabetes; therefore, the condition is also referred to as glucolipotoxicity [152]. Some effects also affect epigenetic mechanisms; for example, the high methylation of the peroxisome proliferator-activated receptor-gamma co-activator 1-alpha gene promoter seen in pancreatic islets of type 2 diabetic individuals results in decreased protein- and glucose-stimulated insulin release [152].

## 6. Recent Advances in Insulin-Signaling Targeted Therapy 

In the treatment of diabetes and nephropathy, numerous aspects of prevention and the multifactorial methodologies used by nephrologists, diabetologists, dieticians, and experienced diabetes specialists to provide a multifaceted care program reduce the progression of kidney diseases. Emerging studies are recommending the employment of the protective properties of metformin against numerous kidney diseases, such as autophagy and AMP-activated protein kinase (AMPK) signaling pathways, to protect the kidneys from injury [153]. Moreover, metformin activates hypoglycemia by decreasing intestinal glucose absorption and hepatic glycogenesis to improve glucose uptake and utilizing peripheral tissues that enhance insulin sensitivity [154]. Another sulfonylurea-receptor-binding drug, sulfonylureas, affects pancreatic β cells, leading to augmented insulin secretion and possibly hypoglycemia [155]. Sodium-glucose co-transporter 2 inhibitors decrease glucose absorption by the kidney, leading to improved glucose excretion and a reduction in hemoglobin A1c of approximately 0.9–1.0% [156]. Thiazolidinediones have been found to improve insulin sensitivity without causing hypoglycemia in their roles as PPARγ agonists, leading to an A1c decrease of 0.5–1.4%, and these drugs, which are metabolized by the liver, are used to treat chronic kidney disease [157]. Alpha-glucosidase inhibitors decrease the breakdown of small intestinal oligo-and disaccharides, reduce the ingestion of carbohydrates, and suspend glucose absorption after a meal [158]. Epidemiological research has shown that resveratrol can provide health benefits, including protection against renal cancer and kidney disease, and its nephroprotective effects have been observed via in vitro and in vivo human and animal studies [159]. Resveratrol has been found to increase AdipoR1 mRNA levels, and its protein expression was eliminated in the presence of FOXO1 shRNA [160]. 

The bioactive agent 3β-Taraxerol is known to affect pancreatic function and acts by enhancing insulin secretion or decreasing intestinal glucose absorption [161]. Gallic acid has been found to reduce circulating levels of TGF-Î^2^1, supporting the hypothesis that it might be used to efficiently manage diabetic nephropathy [162]. The hematological, toxicological, and biochemical effects of orally treating diabetic model mice with 40 mg/kg mangiferin for 30 days were compared to control mice, and the levels of glycosylated hemoglobin, blood glucose, alanine aminotransferase (ALT), aspartate aminotransferase (AST), and alkaline phosphatase were significantly decreased in the mangiferin-treated animals [163]. Alpha-glucosidase inhibitors block carbohydrate absorption in the small intestine [164]. Recently, phytochemicals and components of their signaling pathways have been shown to be effective for prophylaxis and treatment of insulin resistance in GLUT4-expressing tissues. A hydroalcoholic extract of Capparis moonii fruit increased the glucose uptake associated with substantial IRS-1 and IR phosphorylation, PI3-kinase mRNA, and GLUT4 expression in L6 cells [165]. Glucagon-like peptide-1 receptor agonists and diidro-dipeptidyl-peptidase IV inhibitors, which stimulate the incretin system, along with sodium-glucose cotransporter-2 agonists, are the most common classes of antidiabetic drugs used for type 2 diabetes [166]. It was recently revealed that orally administered vanadium complex results in normoglycemia, improves blood SOD, GSH, TP, and LC3 levels, and significantly reduces AST, ALT, TCHO, BUN, MDA, TG, NO, and caspase 3 levels in streptozotocin-induced diabetic mice [167]. Many commercially used antidiabetic drugs (Table 1), including acarbose, miglitol, saxagliptin, dapagliflozin, glimepiride, insulin aspart, bromocriptine, and alogliptin, have various antidiabetic properties and are used to reduce blood glucose levels by improving insulin secretion in type 2 diabetic patients [168].

## 7. Conclusions

Several major leaps in our understanding of diabetes have taken place in clinical practice in recent years. During this time, our knowledge of insulin has developed, and the important roles in the regulation and secretion of insulin in the human body are clear. In this article, we discussed the role of insulin in several physiological processes, including metabolic homeostasis, as well as insulin secretion, regulation, and the development of insulin-signaling targeted therapies. We also provided insights into the role of insulin in the energy balance of several important body organs, thereby extending the horizon of this hormone. However, there is still much that is unknown about insulin, especially in hyperinsulinemia, hypoglycemia, and insulin-signaling targeted therapies. These areas are crucial to insulin regulation, secretion, and the mitigation of insulin-related chronic diseases; therefore, they represent an important suitable road map for future research.

## Figures and Tables

**Table 1:** Protective effects of insulin signaling activators or insulin-signaling targeted therapies against various diseases.

Categories	Drug	Doses	Experimental Model (Human/Animal)	Disease Model	Mode of Action	References
Natural products	Resveratrol	20 µM	Human, mouse	Kidney mesangial cells	Induced AdipoR1 mRNA and protein levels Improved FOXO1 activity	Ji et al. [160]
	Gallic acid	20–40 mg	Rat	Type I diabetic nephropathy	Decreased TGF-β1 levels and creatinine clearance	Garud, and Kulkarni, [162]
	Mangiferin	40 mg	Human	Type 2 diabetes	Reduced blood glucose levels, AST, ALT, and ALP Activated β-catenin	Du et al. [163]
	Saxagliptin	2.5–5 mg	Human, mouse	Type 2 diabetes	Decreased glucagon production Increased insulin production Inhibited DPP-4 activation	Rasouli et al. [168]
	Alpha-glucosidase inhibitors	25 mg		Type 2 diabetes	Reduced renal function Produced hypoglycemic effect	Kumar et al. [169]; Usman et al. [158]
Secondary alcohol	3β-Taraxerol	200 mg	Human	Type 2 diabetes	Improved pancreatic function Increased insulin secretion	Rasouli et al. [161]
Organic compounds	Sulfonylureas	5 mg	Human	Type 2 diabetes	Increased insulin secretion	Nathan et al. [155]
FDA/Commercial Drugs	Metformin	5 mg	Human, mouse, rat	Type 2 diabetes	Improved glucose uptake, lipotoxicity, and antioxidant activities	Pan et al. [153]
	Miglitol	25 mg	Human	Type 2 diabetes	Inhibited α-glucosidase Induced antihyperglycemic activities	Rasouli et al. [168]
	SGLT2 inhibitors	5–10 mg	Human	Type 2 diabetes	Decreased glucose absorption Increased anti-inflammatory and antioxidative activities	Whalen et al. [156]
	Thiazolidinediones	8 mg	Human	Type 2 diabetes	Acted as PPARγ agonist	Soccio et al. [157]
	Colesevelam	3.75 g	Human	Type 2 diabetes	Increased triglycerides Decreased LDLc	Feingold, [170]
	Gallotannins	10–100 ng/mL	Human, rat	Type 2 diabetes	Increased PI3K mRNA and GLUT4 expressions	Kanaujia et al. [165]
	DPP-IV inhibitors	50 mg	Human	Type 2 diabetes	Stimulated incretin system and SGLT-2	Jose and Inzucchi, [166]
	Oxovanadium complex	200 mg	Mouse	Diabetic mice	Increased blood SOD, GSH, TP, and LC3 levels	El-Shafey and Elsherbiny, [167]
	Acarbose	50 mg	human	Type 2 diabetes	Inhibited α-glucosidase and α-amylase	Rasouli et al. [168]

## Data Availability

Not applicable.

## References

[1] Lewis G.F., Brubaker P.L. (2021). The discovery of insulin revisited: Lessons for the modern era. J. Clin. Investig..

[2] Vecchio I., Tornali C., Bragazzi N.L., Martini M. (2018). The Discovery of Insulin: An Important Milestone in the History of Medicine. Front. Endocrinol..

[3] Csajbok E.A., Tamas G. (2016). Cerebral cortex: A target and source of insulin?. Diabetologia.

[4] Alarcon C., Lincoln B., Rhodes C.J. (1993). The biosynthesis of the subtilisin-related proprotein convertase PC3, but no that of the PC2 convertase, is regulated by glucose in parallel to proinsulin biosynthesis in rat pancreatic islets. J. Biol. Chem..

[5] Kaufman B.A., Li C., Soleimanpour S.A. (2015). Mitochondrial regulation of beta-cell function: Maintaining the momentum for insulin release. Mol. Asp. Med..

[6] Vasiljevic J., Torkko J.M., Knoch K.P., Solimena M. (2020). The making of insulin in health and disease. Diabetologia.

[7] Suckale J., Solimena M. (2010). The insulin secretory granule as a signaling hub. Trends Endocrinol. Metab..

[8] Yang B.Y., Zhai G., Gong Y.L., Su J.Z., Peng X.Y., Shang G.H., Han D., Jin J.Y., Liu H.K., Du Z.Y. (2018). Different physiological roles of insulin receptors in mediating nutrient metabolism in zebrafish. Am. J. Physiol. Endocrinol. Metab..

[9] Avruch J. (1998). Insulin signal transduction through protein kinase cascades. Mol. Cell. Biochem..

[10] Taniguchi C.M., Emanuelli B., Kahn C.R. (2006). Critical nodes in signalling pathways: Insights into insulin action. Nat. Rev. Mol. Cell Biol..

[11] Accili D. (2018). Insulin Action Research and the Future of Diabetes Treatment: The 2017 Banting Medal for Scientific Achievement Lecture. Diabetes.

[12] Davidovici B.B., Sattar N., Prinz J., Puig L., Emery P., Barker J.N., van de Kerkhof P., Stahle M., Nestle F.O., Girolomoni G. (2010). Psoriasis and systemic inflammatory diseases: Potential mechanistic links between skin disease and co-morbid conditions. J. Investig. Dermatol..

[13] Plikus M.V., Van Spyk E.N., Pham K., Geyfman M., Kumar V., Takahashi J.S., Andersen B. (2015). The circadian clock in skin: Implications for adult stem cells, tissue regeneration, cancer, aging, and immunity. J. Biol. Rhythm..

[14] Baselga Torres E., Torres-Pradilla M. (2014). Cutaneous manifestations in children with diabetes mellitus and obesity. Actas Dermosifiliogr..

[15] Hough F.S., Pierroz D.D., Cooper C., Ferrari S.L., Bone I.C., Diabetes Working Group (2016). Mechanisms in endocrinology: Mechanisms and evaluation of bone fragility in type 1 diabetes mellitus. Eur. J. Endocrinol..

[16] Hirsch I.B., Juneja R., Beals J.M., Antalis C.J., Wright E.E. (2020). The Evolution of Insulin and How it Informs Therapy and Treatment Choices. Endocr. Rev..

[17] Wendt A., Eliasson L. (2020). Pancreatic alpha-cells—The unsung heroes in islet function. Semin. Cell Dev. Biol.

[18] Cabrera O., Berman D.M., Kenyon N.S., Ricordi C., Berggren P.O., Caicedo A. (2006). The unique cytoarchitecture of human pancreatic islets has implications for islet cell function. Proc. Natl. Acad. Sci. USA.

[19] Rorsman P., Braun M. (2013). Regulation of insulin secretion in human pancreatic islets. Annu. Rev. Physiol..

[20] Jansson L., Hellerstrom C. (1986). Glucose-induced changes in pancreatic islet blood flow mediated by central nervous system. Am. J. Physiol..

[21] Fu Z., Gilbert E.R., Liu D. (2013). Regulation of insulin synthesis and secretion and pancreatic Beta-cell dysfunction in diabetes. Curr. Diabetes Rev..

[22] De Vos A., Heimberg H., Quartier E., Huypens P., Bouwens L., Pipeleers D., Schuit F. (1995). Human and rat beta cells differ in glucose transporter but not in glucokinase gene expression. J. Clin. Investig..

[23] McCulloch L.J., van de Bunt M., Braun M., Frayn K.N., Clark A., Gloyn A.L. (2011). GLUT2 (SLC2A2) is not the principal glucose transporter in human pancreatic beta cells: Implications for understanding genetic association signals at this locus. Mol. Genet. Metab..

[24] Gloyn A.L., Odili S., Zelent D., Buettger C., Castleden H.A., Steele A.M., Stride A., Shiota C., Magnuson M.A., Lorini R. (2005). Insights into the structure and regulation of glucokinase from a novel mutation (V62M), which causes maturity-onset diabetes of the young. J. Biol. Chem..

[25] Doliba N.M., Qin W., Najafi H., Liu C., Buettger C.W., Sotiris J., Collins H.W., Li C., Stanley C.A., Wilson D.F. (2012). Glucokinase activation repairs defective bioenergetics of islets of Langerhans isolated from type 2 diabetics. Am. J. Physiol. Endocrinol. Metab..

[26] Yajima H., Komatsu M., Schermerhorn T., Aizawa T., Kaneko T., Nagai M., Sharp G.W., Hashizume K. (1999). cAMP enhances insulin secretion by an action on the ATP-sensitive K+ channel-independent pathway of glucose signaling in rat pancreatic islets. Diabetes.

[27] Yamada S., Komatsu M., Sato Y., Yamauchi K., Kojima I., Aizawa T., Hashizume K. (2002). Time-dependent stimulation of insulin exocytosis by 3’,5’-cyclic adenosine monophosphate in the rat islet beta-cell. Endocrinology.

[28] Haeusler R.A., McGraw T.E., Accili D. (2018). Biochemical and cellular properties of insulin receptor signalling. Nat. Rev. Mol. Cell Biol..

[29] Saltiel A.R. (2021). Insulin signaling in health and disease. J. Clin. Investig..

[30] Burks D.J., White M.F. (2001). IRS proteins and beta-cell function. Diabetes.

[31] Alves-Bezerra M., Cohen D.E. (2017). Triglyceride Metabolism in the Liver. Compr. Physiol..

[32] Hatting M., Tavares C.D.J., Sharabi K., Rines A.K., Puigserver P. (2018). Insulin regulation of gluconeogenesis. Ann. NY Acad. Sci..

[33] Adeyinka A., Kondamudi N.P. (2021). Hyperosmolar Hyperglycemic Nonketotic Coma. StatPearls [Internet].

[34] Gastaldelli A., Toschi E., Pettiti M., Frascerra S., Quinones-Galvan A., Sironi A.M., Natali A., Ferrannini E. (2001). Effect of physiological hyperinsulinemia on gluconeogenesis in nondiabetic subjects and in type 2 diabetic patients. Diabetes.

[35] Edgerton D.S., Lautz M., Scott M., Everett C.A., Stettler K.M., Neal D.W., Chu C.A., Cherrington A.D. (2006). Insulin’s direct effects on the liver dominate the control of hepatic glucose production. J. Clin. Investig..

[36] Girard J. (2006). Insulin’s effect on the liver: “direct or indirect?” continues to be the question. J. Clin. Investig..

[37] Sharabi K., Tavares C.D., Rines A.K., Puigserver P. (2015). Molecular pathophysiology of hepatic glucose production. Mol. Asp. Med..

[38] Claus T.H., Pilkis S.J. (1976). Regulation by insulin of gluconeogenesis in isolated rat hepatocytes. Biochim. Biophys. Acta.

[39] Marks J.S., Botelho L.H. (1986). Synergistic inhibition of glucagon-induced effects on hepatic glucose metabolism in the presence of insulin and a cAMP antagonist. J. Biol. Chem..

[40] Sindelar D.K., Balcom J.H., Chu C.A., Neal D.W., Cherrington A.D. (1996). A comparison of the effects of selective increases in peripheral or portal insulin on hepatic glucose production in the conscious dog. Diabetes.

[41] Sindelar D.K., Chu C.A., Venson P., Donahue E.P., Neal D.W., Cherrington A.D. (1998). Basal hepatic glucose production is regulated by the portal vein insulin concentration. Diabetes.

[42] Ito K., Maruyama H., Hirose H., Kido K., Koyama K., Kataoka K., Saruta T. (1995). Exogenous insulin dose-dependently suppresses glucopenia-induced glucagon secretion from perfused rat pancreas. Metabolism.

[43] Sindelar D.K., Chu C.A., Rohlie M., Neal D.W., Swift L.L., Cherrington A.D. (1997). The role of fatty acids in mediating the effects of peripheral insulin on hepatic glucose production in the conscious dog. Diabetes.

[44] Obici S., Zhang B.B., Karkanias G., Rossetti L. (2002). Hypothalamic insulin signaling is required for inhibition of glucose production. Nat. Med..

[45] Bergman R.N. (2000). Non-esterified fatty acids and the liver: Why is insulin secreted into the portal vein?. Diabetologia.

[46] Frontera W.R., Ochala J. (2015). Skeletal muscle: A brief review of structure and function. Calcif. Tissue Int..

[47] Baron A.D., Brechtel G., Wallace P., Edelman S.V. (1988). Rates and tissue sites of non-insulin- and insulin-mediated glucose uptake in humans. Am. J. Physiol..

[48] DeFronzo R.A. (2004). Pathogenesis of type 2 diabetes mellitus. Med. Clin. N. Am..

[49] DeFronzo R.A. (1988). Lilly lecture 1987. The triumvirate: Beta-cell, muscle, liver. A collusion responsible for NIDDM. Diabetes.

[50] Defronzo R.A. (2009). Banting Lecture. From the triumvirate to the ominous octet: A new paradigm for the treatment of type 2 diabetes mellitus. Diabetes.

[51] Park S.W., Goodpaster B.H., Lee J.S., Kuller L.H., Boudreau R., de Rekeneire N., Harris T.B., Kritchevsky S., Tylavsky F.A., Nevitt M. (2009). Excessive loss of skeletal muscle mass in older adults with type 2 diabetes. Diabetes Care.

[52] Abdulla H., Smith K., Atherton P.J., Idris I. (2016). Role of insulin in the regulation of human skeletal muscle protein synthesis and breakdown: A systematic review and meta-analysis. Diabetologia.

[53] Romijn J.A., Coyle E.F., Sidossis L.S., Gastaldelli A., Horowitz J.F., Endert E., Wolfe R.R. (1993). Regulation of endogenous fat and carbohydrate metabolism in relation to exercise intensity and duration. Am. J. Physiol..

[54] Taniguchi M., Yoshida H. (2015). Endoplasmic reticulum stress in kidney function and disease. Curr. Opin. Nephrol. Hypertens..

[55] Cheng A., Dube N., Gu F., Tremblay M.L. (2002). Coordinated action of protein tyrosine phosphatases in insulin signal transduction. Eur. J. Biochem..

[56] Karakelides H., Asmann Y.W., Bigelow M.L., Short K.R., Dhatariya K., Coenen-Schimke J., Kahl J., Mukhopadhyay D., Nair K.S. (2007). Effect of insulin deprivation on muscle mitochondrial ATP production and gene transcript levels in type 1 diabetic subjects. Diabetes.

[57] Birsoy K., Festuccia W.T., Laplante M. (2013). A comparative perspective on lipid storage in animals. J. Cell Sci..

[58] Gesta S., Tseng Y.H., Kahn C.R. (2007). Developmental origin of fat: Tracking obesity to its source. Cell.

[59] Smith U. (2002). Impaired (‘diabetic’) insulin signaling and action occur in fat cells long before glucose intolerance--is insulin resistance initiated in the adipose tissue?. Int. J. Obes. Relat. Metab. Disord..

[60] Cignarelli A., Genchi V.A., Perrini S., Natalicchio A., Laviola L., Giorgino F. (2019). Insulin and Insulin Receptors in Adipose Tissue Development. Int. J. Mol. Sci..

[61] Koyama K., Chen G., Lee Y., Unger R.H. (1997). Tissue triglycerides, insulin resistance, and insulin production: Implications for hyperinsulinemia of obesity. Am. J. Physiol..

[62] Favero G., Paganelli C., Buffoli B., Rodella L.F., Rezzani R. (2014). Endothelium and its alterations in cardiovascular diseases: Life style intervention. BioMed Res. Int..

[63] Barac A., Campia U., Panza J.A. (2007). Methods for evaluating endothelial function in humans. Hypertension.

[64] Muniyappa R., Quon M.J. (2007). Insulin action and insulin resistance in vascular endothelium. Curr. Opin. Clin. Nutr. Metab. Care.

[65] Vicent D., Ilany J., Kondo T., Naruse K., Fisher S.J., Kisanuki Y.Y., Bursell S., Yanagisawa M., King G.L., Kahn C.R. (2003). The role of endothelial insulin signaling in the regulation of vascular tone and insulin resistance. J. Clin. Investig..

[66] Potenza M.A., Addabbo F., Montagnani M. (2009). Vascular actions of insulin with implications for endothelial dysfunction. Am. J. Physiol. Endocrinol. Metab..

[67] Hom F.G., Goodner C.J., Berrie M.A. (1984). A [3H]2-deoxyglucose method for comparing rates of glucose metabolism and insulin responses among rat tissues in vivo. Validation of the model and the absence of an insulin effect on brain. Diabetes.

[68] Hasselbalch S.G., Knudsen G.M., Videbaek C., Pinborg L.H., Schmidt J.F., Holm S., Paulson O.B. (1999). No effect of insulin on glucose blood-brain barrier transport and cerebral metabolism in humans. Diabetes.

[69] Havrankova J., Schmechel D., Roth J., Brownstein M. (1978). Identification of insulin in rat brain. Proc. Natl. Acad. Sci. USA.

[70] Ramnanan C.J., Edgerton D.S., Cherrington A.D. (2012). Evidence against a physiologic role for acute changes in CNS insulin action in the rapid regulation of hepatic glucose production. Cell Metab..

[71] Ramnanan C.J., Kraft G., Smith M.S., Farmer B., Neal D., Williams P.E., Lautz M., Farmer T., Donahue E.P., Cherrington A.D. (2013). Interaction between the central and peripheral effects of insulin in controlling hepatic glucose metabolism in the conscious dog. Diabetes.

[72] Benedict C., Hallschmid M., Schultes B., Born J., Kern W. (2007). Intranasal insulin to improve memory function in humans. Neuroendocrinology.

[73] Craft S., Baker L.D., Montine T.J., Minoshima S., Watson G.S., Claxton A., Arbuckle M., Callaghan M., Tsai E., Plymate S.R. (2012). Intranasal insulin therapy for Alzheimer disease and amnestic mild cognitive impairment: A pilot clinical trial. Arch. Neurol..

[74] Taouis M., Torres-Aleman I. (2019). Editorial: Insulin and The Brain. Front. Endocrinol..

[75] Benedict C., Kern W., Schultes B., Born J., Hallschmid M. (2008). Differential sensitivity of men and women to anorexigenic and memory-improving effects of intranasal insulin. J. Clin. Endocrinol. Metab..

[76] Kern W., Benedict C., Schultes B., Plohr F., Moser A., Born J., Fehm H.L., Hallschmid M. (2006). Low cerebrospinal fluid insulin levels in obese humans. Diabetologia.

[77] Whitmer R.A., Gunderson E.P., Barrett-Connor E., Quesenberry C.P., Yaffe K. (2005). Obesity in middle age and future risk of dementia: A 27 year longitudinal population based study. BMJ.

[78] Benedict C., Hallschmid M., Hatke A., Schultes B., Fehm H.L., Born J., Kern W. (2004). Intranasal insulin improves memory in humans. Psychoneuroendocrinology.

[79] Brunner Y.F., Kofoet A., Benedict C., Freiherr J. (2015). Central insulin administration improves odor-cued reactivation of spatial memory in young men. J. Clin. Endocrinol. Metab..

[80] Reger M.A., Watson G.S., Frey W.H., Baker L.D., Cholerton B., Keeling M.L., Belongia D.A., Fishel M.A., Plymate S.R., Schellenberg G.D. (2006). Effects of intranasal insulin on cognition in memory-impaired older adults: Modulation by APOE genotype. Neurobiol. Aging.

[81] Reger M.A., Watson G.S., Green P.S., Wilkinson C.W., Baker L.D., Cholerton B., Fishel M.A., Plymate S.R., Breitner J.C., DeGroodt W. (2008). Intranasal insulin improves cognition and modulates beta-amyloid in early AD. Neurology.

[82] Hallschmid M., Benedict C., Born J., Fehm H.L., Kern W. (2004). Manipulating central nervous mechanisms of food intake and body weight regulation by intranasal administration of neuropeptides in man. Physiol. Behav..

[83] Farber S.J., Berger E.Y., Earle D.P. (1951). Effect of diabetes and insulin of the maximum capacity of the renal tubules to reabsorb glucose. J. Clin. Investig..

[84] Singh S., Sharma R., Kumari M., Tiwari S. (2019). Insulin receptors in the kidneys in health and disease. World J. Nephrol..

[85] Gatica R., Bertinat R., Silva P., Carpio D., Ramirez M.J., Slebe J.C., San Martin R., Nualart F., Campistol J.M., Caelles C. (2013). Altered expression and localization of insulin receptor in proximal tubule cells from human and rat diabetic kidney. J. Cell. Biochem..

[86] Tiwari S., Halagappa V.K., Riazi S., Hu X., Ecelbarger C.A. (2007). Reduced expression of insulin receptors in the kidneys of insulin-resistant rats. J. Am. Soc. Nephrol..

[87] Winzell M.S., Ahren B. (2004). The high-fat diet-fed mouse: A model for studying mechanisms and treatment of impaired glucose tolerance and type 2 diabetes. Diabetes.

[88] Underwood P.C., Adler G.K. (2013). The renin angiotensin aldosterone system and insulin resistance in humans. Curr. Hypertens. Rep..

[89] Csibi A., Communi D., Muller N., Bottari S.P. (2010). Angiotensin II inhibits insulin-stimulated GLUT4 translocation and Akt activation through tyrosine nitration-dependent mechanisms. PLoS ONE.

[90] Akhtar M., Taha N.M., Nauman A., Mujeeb I.B., Al-Nabet A. (2020). Diabetic Kidney Disease: Past and Present. Adv. Anat. Pathol..

[91] Malekzadeh B.O., Erlandsson M.C., Tengvall P., Palmquist A., Ransjo M., Bokarewa M.I., Westerlund A. (2018). Effects of implant-delivered insulin on bone formation in osteoporotic rats. J. Biomed. Mater. Res. A.

[92] Fulzele K., Riddle R.C., DiGirolamo D.J., Cao X., Wan C., Chen D., Faugere M.C., Aja S., Hussain M.A., Bruning J.C. (2010). Insulin receptor signaling in osteoblasts regulates postnatal bone acquisition and body composition. Cell.

[93] Thrailkill K.M., Lumpkin C.K., Bunn R.C., Kemp S.F., Fowlkes J.L. (2005). Is insulin an anabolic agent in bone? Dissecting the diabetic bone for clues. Am. J. Physiol. Endocrinol. Metab..

[94] Xiao Y., Woo W.M., Nagao K., Li W., Terunuma A., Mukouyama Y.S., Oro A.E., Vogel J.C., Brownell I. (2013). Perivascular hair follicle stem cells associate with a venule annulus. J. Investig. Dermatol..

[95] Correll C.U., Robinson D.G., Schooler N.R., Brunette M.F., Mueser K.T., Rosenheck R.A., Marcy P., Addington J., Estroff S.E., Robinson J. (2014). Cardiometabolic risk in patients with first-episode schizophrenia spectrum disorders: Baseline results from the RAISE-ETP study. JAMA Psychiatry.

[96] Pierard G.E., Seite S., Hermanns-Le T., Delvenne P., Scheen A., Pierard-Franchimont C. (2013). The skin landscape in diabetes mellitus. Focus on dermocosmetic management. Clin. Cosmet. Investig. Dermatol..

[97] Napolitano M., Megna M., Monfrecola G. (2015). Insulin resistance and skin diseases. Sci. World J..

[98] Kahana M., Grossman E., Feinstein A., Ronnen M., Cohen M., Millet M.S. (1987). Skin tags: A cutaneous marker for diabetes mellitus. Acta Derm. Venereol..

[99] Cordain L., Lindeberg S., Hurtado M., Hill K., Eaton S.B., Brand-Miller J. (2002). Acne vulgaris: A disease of Western civilization. Arch. Dermatol..

[100] Lindeberg S., Eliasson M., Lindahl B., Ahren B. (1999). Low serum insulin in traditional Pacific Islanders--the Kitava Study. Metabolism.

[101] Melnik B.C., John S.M., Schmitz G. (2011). Over-stimulation of insulin/IGF-1 signaling by western diet may promote diseases of civilization: Lessons learnt from laron syndrome. Nutr. Metab..

[102] Toussirot E., Aubin F., Dumoulin G. (2014). Relationships between Adipose Tissue and Psoriasis, with or without Arthritis. Front. Immunol..

[103] Yadav A., Kataria M.A., Saini V., Yadav A. (2013). Role of leptin and adiponectin in insulin resistance. Clin. Chim. Acta.

[104] Abdel Hay R.M., Rashed L.A. (2011). Association between the leptin gene 2548G/A polymorphism, the plasma leptin and the metabolic syndrome with psoriasis. Exp. Dermatol..

[105] Coimbra S., Oliveira H., Reis F., Belo L., Rocha S., Quintanilha A., Figueiredo A., Teixeira F., Castro E., Rocha-Pereira P. (2010). Circulating adipokine levels in Portuguese patients with psoriasis vulgaris according to body mass index, severity and therapy. J. Eur. Acad. Dermatol. Venereol..

[106] Ruegsegger G.N., Creo A.L., Cortes T.M., Dasari S., Nair K.S. (2018). Altered mitochondrial function in insulin-deficient and insulin-resistant states. J. Clin. Investig..

[107] Thomas D.D., Corkey B.E., Istfan N.W., Apovian C.M. (2019). Hyperinsulinemia: An Early Indicator of Metabolic Dysfunction. J. Endocr. Soc..

[108] Bazotte R.B., Silva L.G., Schiavon F.P. (2014). Insulin resistance in the liver: Deficiency or excess of insulin?. Cell Cycle.

[109] Santoleri D., Titchenell P.M. (2019). Resolving the Paradox of Hepatic Insulin Resistance. Cell. Mol. Gastroenterol. Hepatol..

[110] Titchenell P.M., Quinn W.J., Lu M., Chu Q., Lu W., Li C., Chen H., Monks B.R., Chen J., Rabinowitz J.D. (2016). Direct Hepatocyte Insulin Signaling Is Required for Lipogenesis but Is Dispensable for the Suppression of Glucose Production. Cell Metab..

[111] Ferrannini E. (1998). Insulin resistance versus insulin deficiency in non-insulin-dependent diabetes mellitus: Problems and prospects. Endocr. Rev..

[112] Woo V.C. (2015). New Insulins and New Aspects in Insulin Delivery. Can. J. Diabetes.

[113] Majeed W., Thabit H. (2018). Closed-loop insulin delivery: Current status of diabetes technologies and future prospects. Expert Rev. Med. Devices.

[114] Ramzy A., Mojibian M., Kieffer T.J. (2018). Insulin-Deficient Mouse beta-Cells Do Not Fully Mature but Can Be Remedied Through Insulin Replacement by Islet Transplantation. Endocrinology.

[115] Maneschi F., Mashiter K., Kohner E.M. (1983). Insulin resistance and insulin deficiency in diabetic retinopathy of non-insulin-dependent diabetes. Diabetes.

[116] Cerasi E. (1995). Insulin deficiency and insulin resistance in the pathogenesis of NIDDM: Is a divorce possible?. Diabetologia.

[117] Groop L.C., Widen E., Ferrannini E. (1993). Insulin resistance and insulin deficiency in the pathogenesis of type 2 (non-insulin-dependent) diabetes mellitus: Errors of metabolism or of methods?. Diabetologia.

[118] Kuzuya T., Matsuda A. (1997). Classification of diabetes on the basis of etiologies versus degree of insulin deficiency. Diabetes Care.

[119] Moghetti P., Tosi F. (2021). Insulin resistance and PCOS: Chicken or egg?. J. Endocrinol. Investig..

[120] Thomas N.J., Lynam A.L., Hill A.V., Weedon M.N., Shields B.M., Oram R.A., McDonald T.J., Hattersley A.T., Jones A.G. (2019). Type 1 diabetes defined by severe insulin deficiency occurs after 30 years of age and is commonly treated as type 2 diabetes. Diabetologia.

[121] Kopp W. (2019). How Western Diet and Lifestyle Drive the Pandemic of Obesity and Civilization Diseases. Diabetes Metab. Syndr. Obes..

[122] Jezek P., Jaburek M., Holendova B., Plecita-Hlavata L. (2018). Fatty Acid-Stimulated Insulin Secretion vs. Lipotoxicity. Molecules.

[123] Jia G., Whaley-Connell A., Sowers J.R. (2018). Diabetic cardiomyopathy: A hyperglycaemia- and insulin-resistance-induced heart disease. Diabetologia.

[124] Otto-Buczkowska E., Grzyb K., Jainta N. (2018). Polycystic ovary syndrome (PCOS) and the accompanying disorders of glucose homeostasis among girls at the time of puberty. Pediatr. Endocrinol. Diabetes Metab..

[125] Gerich J.E. (1993). Control of glycaemia. Baillieres Clin. Endocrinol. Metab..

[126] Villegas-Valverde C.C., Kokuina E., Breff-Fonseca M.C. (2018). Strengthening National Health Priorities for Diabetes Prevention and Management. MEDICC Rev..

[127] Hammer M., Storey S., Hershey D.S., Brady V.J., Davis E., Mandolfo N., Bryant A.L., Olausson J. (2019). Hyperglycemia and Cancer: A State-of-the-Science Review. Oncol. Nurs. Forum.

[128] Giugliano D., Ceriello A., Esposito K. (2008). Glucose metabolism and hyperglycemia. Am. J. Clin. Nutr..

[129] Rawlings A.M., Sharrett A.R., Albert M.S., Coresh J., Windham B.G., Power M.C., Knopman D.S., Walker K., Burgard S., Mosley T.H. (2019). The Association of Late-Life Diabetes Status and Hyperglycemia with Incident Mild Cognitive Impairment and Dementia: The ARIC Study. Diabetes Care.

[130] Jacobsen J.J., Black M.H., Li B.H., Reynolds K., Lawrence J.M. (2014). Race/ethnicity and measures of glycaemia in the year after diagnosis among youth with type 1 and type 2 diabetes mellitus. J. Diabetes Complicat..

[131] Yari Z., Behrouz V., Zand H., Pourvali K. (2020). New Insight into Diabetes Management: From Glycemic Index to Dietary Insulin Index. Curr. Diabetes Rev..

[132] Simon K., Wittmann I. (2019). Can blood glucose value really be referred to as a metabolic parameter?. Rev. Endocr. Metab. Disord..

[133] Garber A.J., Cryer P.E., Santiago J.V., Haymond M.W., Pagliara A.S., Kipnis D.M. (1976). The role of adrenergic mechanisms in the substrate and hormonal response to insulin-induced hypoglycemia in man. J. Clin. Investig..

[134] Coutinho M., Gerstein H.C., Wang Y., Yusuf S. (1999). The relationship between glucose and incident cardiovascular events. A metaregression analysis of published data from 20 studies of 95,783 individuals followed for 12.4 years. Diabetes Care.

[135] Decode Study Group, European Diabetes Epidemiology Group (2003). Is the current definition for diabetes relevant to mortality risk from all causes and cardiovascular and noncardiovascular diseases?. Diabetes Care.

[136] Gosmanov A.R., Gosmanova E.O., Kitabchi A.E., Feingold K.R., Anawalt B., Boyce A., Chrousos G., de Herder W.W., Dhatariya K., Dungan K., Grossman A., Hershman J.M., Hofland J. (2000). Hyperglycemic Crises: Diabetic Ketoacidosis and Hyperglycemic Hyperosmolar State. Endotext.

[137] Rahman M.S., Adegoke E.O., Pang M.G. (2021). Drivers of owning more BPA. J. Hazard. Mater..

[138] Esposito K., Giugliano D. (2006). Diet and inflammation: A link to metabolic and cardiovascular diseases. Eur. Heart J..

[139] Gorska E., Popko K., Stelmaszczyk-Emmel A., Ciepiela O., Kucharska A., Wasik M. (2010). Leptin receptors. Eur. J. Med. Res..

[140] Marroqui L., Gonzalez A., Neco P., Caballero-Garrido E., Vieira E., Ripoll C., Nadal A., Quesada I. (2012). Role of leptin in the pancreatic beta-cell: Effects and signaling pathways. J. Mol. Endocrinol..

[141] Lumeng C.N., Saltiel A.R. (2011). Inflammatory links between obesity and metabolic disease. J. Clin. Investig..

[142] Khodabandehloo H., Gorgani-Firuzjaee S., Panahi G., Meshkani R. (2016). Molecular and cellular mechanisms linking inflammation to insulin resistance and beta-cell dysfunction. Transl. Res..

[143] Boni-Schnetzler M., Boller S., Debray S., Bouzakri K., Meier D.T., Prazak R., Kerr-Conte J., Pattou F., Ehses J.A., Schuit F.C. (2009). Free fatty acids induce a proinflammatory response in islets via the abundantly expressed interleukin-1 receptor I. Endocrinology.

[144] Tang C., Naassan A.E., Chamson-Reig A., Koulajian K., Goh T.T., Yoon F., Oprescu A.I., Ghanim H., Lewis G.F., Dandona P. (2013). Susceptibility to fatty acid-induced beta-cell dysfunction is enhanced in prediabetic diabetes-prone biobreeding rats: A potential link between beta-cell lipotoxicity and islet inflammation. Endocrinology.

[145] McGarry J.D., Dobbins R.L. (1999). Fatty acids, lipotoxicity and insulin secretion. Diabetologia.

[146] Boden G., Shulman G.I. (2002). Free fatty acids in obesity and type 2 diabetes: Defining their role in the development of insulin resistance and beta-cell dysfunction. Eur. J. Clin. Investig..

[147] Shimabukuro M., Wang M.Y., Zhou Y.T., Newgard C.B., Unger R.H. (1998). Protection against lipoapoptosis of beta cells through leptin-dependent maintenance of Bcl-2 expression. Proc. Natl. Acad. Sci. USA.

[148] Gravena C., Mathias P.C., Ashcroft S.J. (2002). Acute effects of fatty acids on insulin secretion from rat and human islets of Langerhans. J. Endocrinol..

[149] Yamashita T., Eto K., Okazaki Y., Yamashita S., Yamauchi T., Sekine N., Nagai R., Noda M., Kadowaki T. (2004). Role of uncoupling protein-2 up-regulation and triglyceride accumulation in impaired glucose-stimulated insulin secretion in a beta-cell lipotoxicity model overexpressing sterol regulatory element-binding protein-1c. Endocrinology.

[150] Joseph J.W., Koshkin V., Saleh M.C., Sivitz W.I., Zhang C.Y., Lowell B.B., Chan C.B., Wheeler M.B. (2004). Free fatty acid-induced beta-cell defects are dependent on uncoupling protein 2 expression. J. Biol. Chem..

[151] Lupi R., Dotta F., Marselli L., Del Guerra S., Masini M., Santangelo C., Patane G., Boggi U., Piro S., Anello M. (2002). Prolonged exposure to free fatty acids has cytostatic and pro-apoptotic effects on human pancreatic islets: Evidence that beta-cell death is caspase mediated, partially dependent on ceramide pathway, and Bcl-2 regulated. Diabetes.

[152] Poitout V., Robertson R.P. (2008). Glucolipotoxicity: Fuel excess and beta-cell dysfunction. Endocr. Rev..

[153] Pan Q., Lu X., Zhao C., Liao S., Chen X., Guo F., Yang C., Liu H.F. (2020). Metformin: The updated protective property in kidney disease. Aging.

[154] Zhou T., Xu X., Du M., Zhao T., Wang J. (2018). A preclinical overview of metformin for the treatment of type 2 diabetes. Biomed. Pharmacother..

[155] Nathan D.M., Buse J.B., Davidson M.B., Ferrannini E., Holman R.R., Sherwin R., Zinman B. (2009). Medical management of hyperglycemia in type 2 diabetes: A consensus algorithm for the initiation and adjustment of therapy: A consensus statement of the American Diabetes Association and the European Association for the Study of Diabetes. Diabetes Care.

[156] Whalen K., Miller S., Onge E.S. (2015). The Role of Sodium-Glucose Co-Transporter 2 Inhibitors in the Treatment of Type 2 Diabetes. Clin. Ther..

[157] Soccio R.E., Chen E.R., Lazar M.A. (2014). Thiazolidinediones and the Promise of Insulin Sensitization in Type 2 Diabetes. Cell Metab..

[158] Usman B., Sharma N., Satija S., Mehta M., Vyas M., Khatik G.L., Khurana N., Hansbro P.M., Williams K., Dua K. (2019). Recent Developments in Alpha-Glucosidase Inhibitors for Management of Type-2 Diabetes: An Update. Curr. Pharm. Des..

[159] Den Hartogh D.J., Tsiani E. (2019). Health Benefits of Resveratrol in Kidney Disease: Evidence from In Vitro and In Vivo Studies. Nutrients.

[160] Ji H., Wu L., Ma X., Ma X., Qin G. (2014). The effect of resveratrol on the expression of AdipoR1 in kidneys of diabetic nephropathy. Mol. Biol. Rep..

[161] Yang S.C., Hsu C.Y., Chou W.L., Fang J.Y., Chuang S.Y. (2020). Bioactive Agent Discovery from the Natural Compounds for the Treatment of Type 2 Diabetes Rat Model. Molecules.

[162] Garud M.S., Kulkarni Y.A. (2018). Gallic acid attenuates type I diabetic nephropathy in rats. Chem. Biol. Interact..

[163] Du S.Y., Liu H.R., Lei T.T., Xie X.F., Wang H.L., He X., Tong R.S., Wang Y. (2018). Mangiferin: An effective therapeutic agent against several disorders. Mol. Med. Rep..

[164] Yen F.S., Wei J.C., Lin M.C., Hsu C.C., Hwu C.M. (2021). Long-term outcomes of adding alpha-glucosidase inhibitors in insulin-treated patients with type 2 diabetes. BMC Endocr. Disord..

[165] Kanaujia A., Duggar R., Pannakal S.T., Yadav S.S., Katiyar C.K., Bansal V., Anand S., Sujatha S., Lakshmi B.S. (2010). Insulinomimetic activity of two new gallotannins from the fruits of Capparis moonii. Bioorg. Med. Chem..

[166] Jose T., Inzucchi S.E. (2012). Cardiovascular effects of the DPP-4 inhibitors. Diabetes Vasc. Dis. Res..

[167] El-Shafey E.S., Elsherbiny E.S. (2020). The role of apoptosis and autophagy in the insulin-enhancing activity of oxovanadium(IV) bipyridine complex in streptozotocin-induced diabetic mice. Biometals.

[168] Rasouli H., Yarani R., Pociot F., Popovic-Djordjevic J. (2020). Anti-diabetic potential of plant alkaloids: Revisiting current findings and future perspectives. Pharmacol. Res..

[169] Kumar Y., Thakur A.K., Goyal R.K. (2019). Evaluation of Alpha Glucosidase Inhibitor (Miglitol) for its Efficacy in Constipation Associated with Diabetes. Indian J. Pharm. Educ..

[170] Feingold K.R., Feingold K.R., Anawalt B., Boyce A., Chrousos G., de Herder W.W., Dhatariya K., Dungan K., Grossman A., Hershman J.M., Hofland J. (2000). Oral and Injectable (Non-Insulin) Pharmacological Agents for Type 2 Diabetes. Endotext.

